# Supplementary material for: Causal Relationship between Aging and Anorexia Nervosa: A White-Matter-Microstructure-Mediated Mendelian Randomization Analysis
Source: Biomedicines. 2024 Aug 16;12(8):1874. doi: 10.3390/biomedicines12081874 (PMC11351342; doi:10.3390/biomedicines12081874)
Supplement: Supplementary file 1 [file biomedicines-12-01874-s001.zip › Supplementary Figure/Figure S4/Singe SNP plot of MR analysis for TL to ubm-b-1814.pdf]

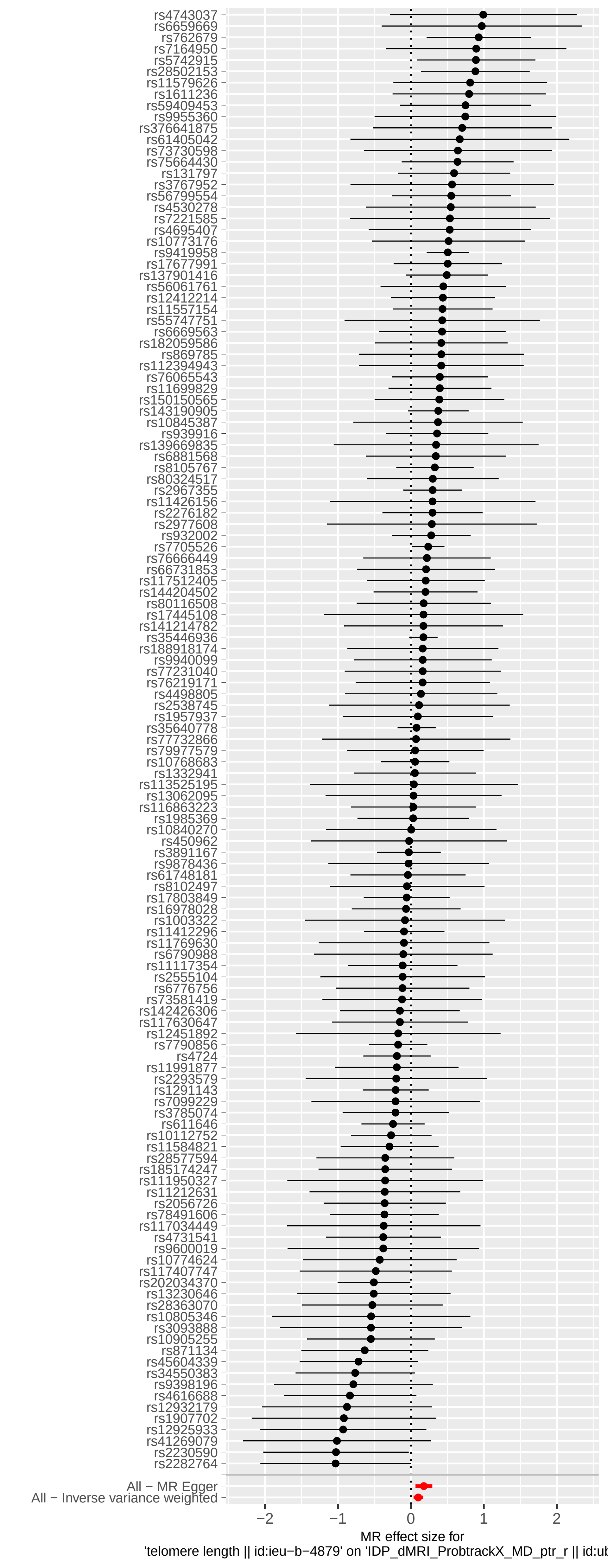
rs4743037
rs6659669
rs762679
rs7164950
rs5742915
rs28502153
rs11579626
rs1611236
rs59409453
rs9955360
rs376641875
rs61405042
rs73730598
rs75664430
rs131797
rs3767952
rs56799554
rs4530278
rs7221585
rs4695407
rs10773176
rs9419958
rs17677991
rs137901416
rs56061761
rs12412214
rs11557154
rs55747751
rs6669563
rs182059586
rs869785
rs112394943
rs76065543
rs11699829
rs150150565
rs143190905
rs10845387
rs939916
rs139669835
rs6881568
rs8105767
rs80324517
rs2967355
rs11426156
rs2276182
rs2977608
rs932002
rs7705526
rs76666449
rs66731853
rs117512405
rs144204502
rs80116508
rs17445108
rs141214782
rs35446936
rs188918174
rs9940099
rs77231040
rs76219171
rs4498805
rs2538745
rs1957937
rs35640778
rs77732866
rs79977579
rs10768683
rs1332941
rs113525195
rs13062095
rs116863223
rs1985369
rs10840270
rs450962
rs3891167
rs9878436
rs61748181
rs8102497
rs17803849
rs16978028
rs1003322
rs11412296
rs11769630
rs6790988
rs11117354
rs2555104
rs6776756
rs73581419
rs142426306
rs117630647
rs12451892
rs7790856
rs4724
rs11991877
rs2293579
rs1291143
rs7099229
rs3785074
rs611646
rs10112752
rs11584821
rs28577594
rs185174247
rs111950327
rs11212631
rs2056726
rs78491606
rs117034449
rs4731541
rs9600019
rs10774624
rs117407747
rs202034370
rs13230646
rs28363070
rs10805346
rs3093888
rs10905255
rs871134
rs45604339
rs34550383
rs9398196
rs4616688
rs12932179
rs1907702
rs12925933
rs41269079
rs2230590
rs2282764
All − MR Egger
All − Inverse variance weighted
−2
−1
0
1
2
MR effect size for
'telomere length || id:ieu−b−4879' on 'IDP_dMRI_ProbtrackX_MD_ptr_r || id:ub
